# Supplementary material for: Unveiling the Bioactive Potential of the Invasive Jellyfish Phyllorhiza punctata Through Integrative Transcriptomic and Proteomic Analyses
Source: Biomolecules. 2025 Aug 4;15(8):1121. doi: 10.3390/biom15081121 (PMC12383608; doi:10.3390/biom15081121)
Supplement: Supplementary file 1 [file biomolecules-15-01121-s001.zip › Suplementary Material/FileS3/PEPFOLD4/XP_065062718_8656/clusters.html]

 PEP-FOLD clusters


Download archive of all models  here   

|  |  |  |
| --- | --- | --- |
| # | file | sOPEP |
| # cluster 1 - Size: 25 |  |  |
|  | XP\_065062718\_8656-model1 | -15.0394 |
|  | XP\_065062718\_8656-model1.1 | -15.0394 |
|  | XP\_065062718\_8656-model1.2 | -14.9734 |
|  | XP\_065062718\_8656-model1.3 | -14.9668 |
|  | XP\_065062718\_8656-model1.4 | -14.8557 |
|  | XP\_065062718\_8656-model1.5 | -14.8557 |
|  | XP\_065062718\_8656-model1.6 | -14.8557 |
|  | XP\_065062718\_8656-model1.7 | -14.8523 |
|  | XP\_065062718\_8656-model1.8 | -14.8339 |
|  | XP\_065062718\_8656-model1.9 | -14.8229 |
|  | XP\_065062718\_8656-model1.10 | -14.8207 |
|  | XP\_065062718\_8656-model1.11 | -14.8175 |
|  | XP\_065062718\_8656-model1.12 | -14.8175 |
|  | XP\_065062718\_8656-model1.13 | -14.8038 |
|  | XP\_065062718\_8656-model1.14 | -14.7865 |
|  | XP\_065062718\_8656-model1.15 | -14.7749 |
|  | XP\_065062718\_8656-model1.16 | -14.7443 |
|  | XP\_065062718\_8656-model1.17 | -14.6927 |
|  | XP\_065062718\_8656-model1.18 | -14.6927 |
|  | XP\_065062718\_8656-model1.19 | -14.6927 |
|  | XP\_065062718\_8656-model1.20 | -14.6847 |
|  | XP\_065062718\_8656-model1.21 | -14.6594 |
|  | XP\_065062718\_8656-model1.22 | -14.6458 |
|  | XP\_065062718\_8656-model1.23 | -14.6458 |
|  | XP\_065062718\_8656-model1.24 | -14.5941 |
| # cluster 2 - Size: 46 |  |  |
|  | XP\_065062718\_8656-model2 | -14.9334 |
|  | XP\_065062718\_8656-model2.1 | -14.8943 |
|  | XP\_065062718\_8656-model2.2 | -14.8943 |
|  | XP\_065062718\_8656-model2.3 | -14.8943 |
|  | XP\_065062718\_8656-model2.4 | -14.8589 |
|  | XP\_065062718\_8656-model2.5 | -14.8589 |
|  | XP\_065062718\_8656-model2.6 | -14.8589 |
|  | XP\_065062718\_8656-model2.7 | -14.8458 |
|  | XP\_065062718\_8656-model2.8 | -14.8458 |
|  | XP\_065062718\_8656-model2.9 | -14.8451 |
|  | XP\_065062718\_8656-model2.10 | -14.8451 |
|  | XP\_065062718\_8656-model2.11 | -14.8451 |
|  | XP\_065062718\_8656-model2.12 | -14.8451 |
|  | XP\_065062718\_8656-model2.13 | -14.7959 |
|  | XP\_065062718\_8656-model2.14 | -14.7959 |
|  | XP\_065062718\_8656-model2.15 | -14.7959 |
|  | XP\_065062718\_8656-model2.16 | -14.7959 |
|  | XP\_065062718\_8656-model2.17 | -14.7842 |
|  | XP\_065062718\_8656-model2.18 | -14.7363 |
|  | XP\_065062718\_8656-model2.19 | -14.7363 |
|  | XP\_065062718\_8656-model2.20 | -14.7363 |
|  | XP\_065062718\_8656-model2.21 | -14.7363 |
|  | XP\_065062718\_8656-model2.22 | -14.7259 |
|  | XP\_065062718\_8656-model2.23 | -14.7259 |
|  | XP\_065062718\_8656-model2.24 | -14.7259 |
|  | XP\_065062718\_8656-model2.25 | -14.7259 |
|  | XP\_065062718\_8656-model2.26 | -14.7259 |
|  | XP\_065062718\_8656-model2.27 | -14.7082 |
|  | XP\_065062718\_8656-model2.28 | -14.7082 |
|  | XP\_065062718\_8656-model2.29 | -14.7082 |
|  | XP\_065062718\_8656-model2.30 | -14.7082 |
|  | XP\_065062718\_8656-model2.31 | -14.7082 |
|  | XP\_065062718\_8656-model2.32 | -14.7082 |
|  | XP\_065062718\_8656-model2.33 | -14.6964 |
|  | XP\_065062718\_8656-model2.34 | -14.6964 |
|  | XP\_065062718\_8656-model2.35 | -14.6964 |
|  | XP\_065062718\_8656-model2.36 | -14.6964 |
|  | XP\_065062718\_8656-model2.37 | -14.6964 |
|  | XP\_065062718\_8656-model2.38 | -14.6964 |
|  | XP\_065062718\_8656-model2.39 | -14.689 |
|  | XP\_065062718\_8656-model2.40 | -14.689 |
|  | XP\_065062718\_8656-model2.41 | -14.689 |
|  | XP\_065062718\_8656-model2.42 | -14.6851 |
|  | XP\_065062718\_8656-model2.43 | -14.6851 |
|  | XP\_065062718\_8656-model2.44 | -14.6335 |
|  | XP\_065062718\_8656-model2.45 | -14.5729 |
| # cluster 3 - Size: 22 |  |  |
|  | XP\_065062718\_8656-model3 | -14.9192 |
|  | XP\_065062718\_8656-model3.1 | -14.8591 |
|  | XP\_065062718\_8656-model3.2 | -14.8263 |
|  | XP\_065062718\_8656-model3.3 | -14.8263 |
|  | XP\_065062718\_8656-model3.4 | -14.8263 |
|  | XP\_065062718\_8656-model3.5 | -14.8263 |
|  | XP\_065062718\_8656-model3.6 | -14.8263 |
|  | XP\_065062718\_8656-model3.7 | -14.8118 |
|  | XP\_065062718\_8656-model3.8 | -14.7459 |
|  | XP\_065062718\_8656-model3.9 | -14.7459 |
|  | XP\_065062718\_8656-model3.10 | -14.7424 |
|  | XP\_065062718\_8656-model3.11 | -14.7133 |
|  | XP\_065062718\_8656-model3.12 | -14.6848 |
|  | XP\_065062718\_8656-model3.13 | -14.6771 |
|  | XP\_065062718\_8656-model3.14 | -14.6771 |
|  | XP\_065062718\_8656-model3.15 | -14.6626 |
|  | XP\_065062718\_8656-model3.16 | -14.619 |
|  | XP\_065062718\_8656-model3.17 | -14.619 |
|  | XP\_065062718\_8656-model3.18 | -14.6095 |
|  | XP\_065062718\_8656-model3.19 | -14.6095 |
|  | XP\_065062718\_8656-model3.20 | -14.5837 |
|  | XP\_065062718\_8656-model3.21 | -14.5794 |
| # cluster 4 - Size: 6 |  |  |
|  | XP\_065062718\_8656-model4 | -14.801 |
|  | XP\_065062718\_8656-model4.1 | -14.801 |
|  | XP\_065062718\_8656-model4.2 | -14.7717 |
|  | XP\_065062718\_8656-model4.3 | -14.7717 |
|  | XP\_065062718\_8656-model4.4 | -14.7026 |
|  | XP\_065062718\_8656-model4.5 | -14.6411 |
| # cluster 5 - Size: 1 |  |  |
|  | XP\_065062718\_8656-model5 | -14.581 |
|  |  |  |
